# Successful treatment of highly advanced immunoglobulin G4-related kidney disease presenting renal mass-like regions with end-stage kidney failure: a case study

**DOI:** 10.1186/s12882-017-0676-5

**Published:** 2017-08-03

**Authors:** Hiroyuki Ono, Taichi Murakami, Akira Mima, Eriko Shibata, Masanori Tamaki, Sakiya Yoshimoto, Sayo Ueda, Fumi Kishi, Seiji Kishi, Takashi Kawanaka, Motokazu Matsuura, Kojiro Nagai, Hideharu Abe, Masashi Harada, Toshio Doi

**Affiliations:** 1 Department of Nephrology, Tokushima University Graduate School, 3-18-15 Kuramoto, Tokushima, Tokushima 770-8503 Japan; 2 Department of Nephrology, Nara Hospital, Kindai University Faculty of Medicine, 1248-1 Otoda-cho, Ikoma, Nara, 630-0293 Japan; 3 Department of Radiology, Tokushima University Graduate School, 3-18-15 Kuramoto, Tokushima, Tokushima 770-8503 Japan

**Keywords:** Immunoglobulin G4, End-stage kidney disease, Tubulointerstitial nephritis, Fibrosis

## Abstract

**Background:**

Immunoglobulin G4-related kidney disease characterized by immunoglobulin G4-positive plasma cell-rich tubulointerstitial nephritis has distinctive serological and radiological findings. Renal prognosis is good because of a good response to glucocorticoids. Here we report a case of successful treatment of highly advanced immunoglobulin G4-related kidney disease presenting renal mass-like regions with end-stage kidney failure.

**Case Presentation:**

A 59-year-old Japanese man was referred to our hospital because of uremia with a creatinine level of 12.36 mg/dL. Urinalysis revealed mild proteinuria and hyperβ2microglobulinuria, and blood tests showed hyperglobulinemia with an IgG level of 3243 mg/dL and an IgG4 level of 621 mg/dL. Non-contrast computed tomography revealed renal mass-like regions. Based on the findings, immunoglobulin G4-related kidney disease was suspected, however, further radiological examination showed unexpected results. Ga-67 scintigraphy showed no kidney uptake. T2-weighted magnetic resonance imaging revealed high-intensity signals which corresponded to mass-like regions and multiple patchy low-intensity signals in kidney cortex. Finally, the patient was diagnosed with immunoglobulin G4-related kidney disease by renal pathology of severe immunoglobulin G4-positive plasma cell-rich tubulointerstitial nephritis and characteristic fibrosis. He received 50 mg oral prednisolone, which was tapered with a subsequent decrease of serum creatinine and IgG4 levels. One year after initiation of treatment, he achieved normalization of serum IgG4 level and proteinuria, and remained off dialysis with a creatinine level of 3.50 mg/dL. After treatment with steroids, repeat imaging suggested bilateral severe focal atrophy. However, mass-like regions did not show atrophic change although renal atrophy was evident in patchy low-intensity lesions on T2-weighted magnetic resonance imaging. These findings suggest that multiple patchy low-intensity signals and high-intensity mass-like regions were mildly atrophic lesions of immunoglobulin G4-related kidney disease due to severe fibrosis and normal parts of kidney, respectively.

**Conclusions:**

In immunoglobulin G4-related kidney disease with severe kidney failure, radiological findings should be carefully examined. In addition, renal prognosis may be good despite highly advanced tubulointerstitial nephritis and fibrosis.

## Background

IgG4-related disease (IgG4-RD) is a recently recognized emerging clinicopathological entity characterized by several features: tumefactive lesions, a dense lymphoplasmacytic infiltrate rich in IgG4-positive plasma cells with fibrosis, and usually an elevated serum IgG4 concentration [1]. This fibro-inflammatory condition occurs in multiple organs including the kidneys. Clinical symptoms vary depending on the affected organ, but they are relatively mild, and organ swelling may be the only diagnostic clue in many patients. IgG4-RD is diagnosed by a combination of clinical serological and radiological findings along with pathological features [2]. IgG4-related kidney disease (IgG4-RKD) is a comprehensive term for renal lesions associated with IgG4-RD [3]. Here, we describe a case of highly advanced IgG4-RKD presenting mass-like regions with end-stage kidney failure.

## Case presentation

**Fig. 1:**
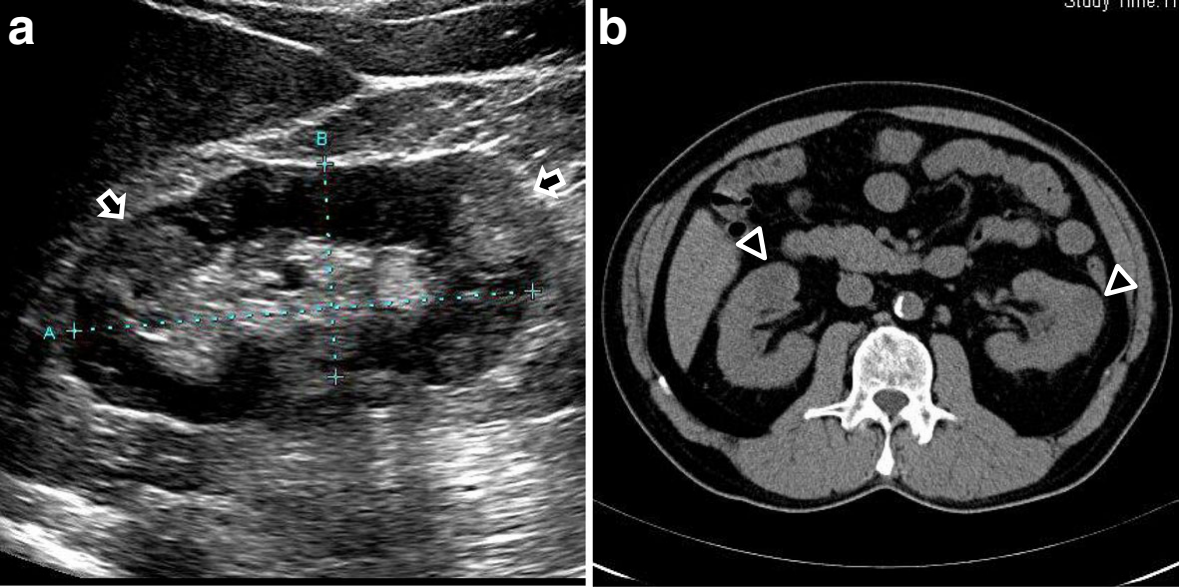
Findings of Echography and computed tomography before treatment. (**a**) Multiple high echoic lesions (*arrows*) by Echography and (**b**) mass-like regions (*arrow head*) by computed tomography

**Fig. 2:**
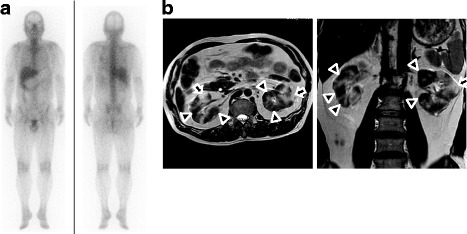
Findings of Ga-67 scintigraphy and MR imaging. (**a**) No uptake to kidney by Ga-67 scintigraphy and (**b**) low-intensity signals (*arrow head*) and high-intensity signals (*arrow*) by T2-weighted MR imaging

**Fig. 3:**
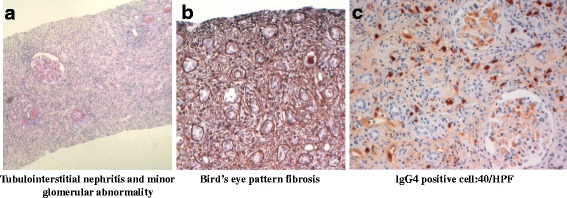
Pathological findings. (**a**) Tubulointerstitial nephritis and minor glomerular abnormality shown by PAS stain, (**b**) Bird’s eye pattern fibrosis shown by PAM stain and (**c**) IgG4 positive cell:40/HPF shown by Immunostaining for IgG4

**Fig. 4:**
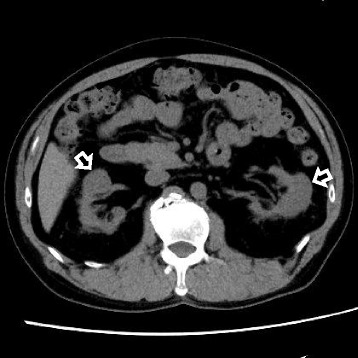
Findings of computed tomography after treatment. Focal atrophy of kidneys. Mass-like regions before treatment did not show atrophic change by computed tomography

A 59-year-old man was admitted to our hospital because of severe kidney failure. He complained only of general fatigue. He had no medical history and was on no medication. Urinalysis revealed no hematuria, mild proteinuria (Total protein 0.56 g/day) and hyperβ2microglobulinuria although he had no abnormal urinary findings until 6 months prior to admission. Blood tests showed anemia with a hemoglobin level of 10.5 g/dL, kidney failure with a creatinine level of 12.36 mg/dL (normal level, 0.5–1.1 mg/dL), hypocomplementemia with a C3 level of 51 mg/dL and C4 level of 3 mg/dL, an elevated serum anti-double-stranded DNA antibody level of 44 IU/mL, and hyperglobulinemia with an IgG level of 3243 mg/dL (normal level, 870–1700 mg/dL) and an IgG4 level of 621 mg/dL (normal level, 4.8–105 mg/dL). Echography and computed tomography without contrast revealed mass-like regions (Fig. 1). Based on these findings, tumefactive lesion of IgG4-RKD was suspected, but no other intercurrent IgG4-related lesion such as pancreatitis was noted. Further imaging examinations were performed to detect diagnostic findings associated with IgG4-RKD. Ga-67 scintigraphy showed no kidney uptake. T2-weighted magnetic resonance (MR) imaging revealed multiple patchy low-intensity signals and some high-intensity signals in kidney cortex. High-intensity signals corresponded to mass-like regions (Fig. 2). These findings were not typical for IgG4-RKD. Finally, he was diagnosed with IgG4-RKD by renal pathology with massive tubulointerstitial nephritis (TIN), characteristic fibrosis (bird’s eye pattern), and IgG4-positive plasma cell infiltrate (Fig. 3). There was no deposition of globulin or complement in the glomeruli and no evidence of glomerular sclerosis. He received 50 mg oral prednisolone. With a subsequent decrease of serum creatinine and IgG4 levels, prednisolone was decreased by 2.5 to 10 mg every two to four weeks after induction therapy for 6 weeks. Finally he received maintenance therapy with 5 mg of prednisolone 6 months after initiation of treatment. One year after initiation of treatment, he achieved normalization of serum IgG4 level and proteinuria, and remained off dialysis with a creatinine level of 3.50 mg/dL, although severe focal atrophy developed in bilateral kidney (Fig. 4). However, mass-like regions did not show atrophic change although renal atrophy was evident in patchy low-intensity lesions on T2-weighted magnetic resonance imaging.

## Discussion and conclusions

IgG4-RD predominantly affects middle-aged and older men. Most patients with IgG4-RKD have IgG4-related extrarenal lesions such as in the salivary glands, lacrimal glands, lymph nodes, or pancreas. Clinical symptoms differ depending on the involved organs [4]. The most dominant features of IgG4-RKD are IgG4-positive plasma cell-rich TIN and characteristic fibrosis, which are sometimes concurrent with glomerular lesions [3, 5]. Abnormal urinary findings and clinical symptoms specific to IgG4-related TIN are mild, although edema caused by glomerular lesions or hydronephrosis may be a diagnostic clue. Thus, IgG4-RKD is often diagnosed during examination of a systemic IgG4-related disease. However, our patient had no extrarenal lesions and no evidence of physical findings, so the final diagnostic clue was uremia with general fatigue resulting from severe kidney failure. Urinary findings showed only mild proteinuria and hyperβ2microglobulinuria. Renal pathology revealed severe TIN with fibrosis specific to IgG4-RKD and minor glomerular abnormalities. These data suggest that the patient’s end stage kidney failure resulted from IgG4-related TIN.

A definitive diagnosis of IgG4-RKD is achieved mainly by laboratory data and renal pathology, but characteristic radiological findings are also useful: low-density lesions by enhanced computed tomography, increased uptake by Ga-67 scintigraphy and FDG-PET, and low- and high-intensity lesions on T2-weighted and diffusion-weighted MR imaging [6, 7]. These findings indicate a hypo-vascular free water-eliminated lesion with dense cellular infiltration and fibrosis of IgG4-TIN. Our patient showed atypical radiological findings for IgG4-RKD: multiple renal cortex mass-like regions which show high-intensity signals on T2-weighted MR imaging and no uptake to kidney by Ga-67 scintigraphy. After treatment, mass-like regions did not show atrophic change although renal atrophy was evident in low-intensity lesions on T2-weighted MR imaging. Kidney parenchymal lesions were categorized as follows: patchy or wedged-shaped lesions, diffuse lesions or peripheral cortical nodules [7]. Patchy lesions show convex shape when TIN is severe. Patients with diffuse lesions often show swollen kidney with decreased kidney function. These findings suggest that our patient before treatment showed end stage kidney disease with mild focal kidney atrophy due to highly advanced multiple lesions of IgG4-TIN. Therefore, normal parts of kidney looked “mass-like regions” in this case although IgG4-RKD presents tumefactive lesions in some cases. In an IgG4-RKD patient with advanced kidney failure, the imaging tests should be carefully examined.

The optimal treatment for IgG4-RD has not been established, but most patients respond to glucocorticoid therapy [1]. In a study of 43 Japanese patients with IgG4-RKD, glucocorticoid treatment led a rapid improvement of kidney function and serological abnormalities 1 month after the start of therapy, and maintenance therapy with low-dose glucocorticoids suppressed disease activity for a relatively long time [4]. However, one patient with advanced kidney damage due to IgG4-TIN required maintenance hemodialysis. Renal atrophy after treatment frequently occurs, especially in patients with advanced kidney damage. Atrophic changes probably resulted from advanced fibrosis and decreased infiltration of inflammatory cell in the renal interstitium. These findings suggest that early diagnosis and treatment of IgG4-TIN are essential. Our patient had end-stage kidney disease with an estimated glomerular filtration rate (eGFR) < 10 mL/min at the time of diagnosis and finally had severe focal renal atrophy. However, he did not require maintenance dialysis, as his eGFR was about 20 mL/min while undergoing maintenance therapy. The clinical cause of his favorable outcome may be that he had no complications such as hypertension or diabetes mellitus, and did not take a renal toxic reagent such as contrast. The pathological features of IgG4-TIN may also contribute to the outcome. Nephron function in the non-fibrotic region is probably maintained in IgG4-related TIN because plasma cell-infiltrative and fibrotic lesions show a well-defined regional distribution. In this case, nephron probably functions in “mass-like” regions. It may be also favorable that necrotizing angiitis and advanced tubulitis, which may decrease kidney function, are rare in IgG4-TIN in contrast to other types of TIN caused by a drug or collagen disease [8]. Taken together, IgG4-related TIN should be actively treated with glucocorticoids even though kidney function greatly decreases at the time of diagnosis.

IgG4-RKD is a recently recognized condition with pathological feature. Renal prognosis is relatively good because, in general, patients respond rapidly to glucocorticoids. The clinical evidence for IgG4-RKD is still being investigated. Our case adds evidence to the pathophysiology of IgG4-RKD.

## Abbreviations

eGFR: Estimated glomerular filtration rate
IgG4-RD: IgG4-related disease
IgG4-RKD: IgG4-related kidney disease
MR: Magnetic resonance
TIN: Tubulointerstitial nephritis
